# Clinical Distribution and Virulence Gene Profiles of CNF1- and CNF2-Positive *Escherichia coli* in Calves

**DOI:** 10.3390/ijms27219408

**Published:** 2026-10-22

**Authors:** Michał Bednarski, Robert Kupczyński

**Affiliations:** 1 Department of Epizootiology and Clinic of Birds and Exotic Animals, Faculty of Veterinary Medicine, Wroclaw University of Environmental and Life Sciences, 50-366 Wroclaw, Poland; 2 Department of Environmental Hygiene and Animal Welfare, Faculty of Biology and Animal Science, Wroclaw University of Environmental and Life Sciences, 51-630 Wroclaw, Poland; robert.kupczynski@upwr.edu.pl

**Keywords:** necrotoxigenic *Escherichia coli* (NTEC), CNF1, CNF2, virulence genes, ExPEC, K1 capsule, calves

## Abstract

Cytotoxic necrotizing factor (CNF)-associated *Escherichia coli* (*E. coli*) occur in enteric and extraintestinal disease in calves, but the distribution of CNF1 and CNF2 and their virulence profiles remain incompletely defined. We analysed *E. coli* from 544 calves: 131 healthy, 210 diarrhoeic, 173 with septicaemia, and 30 with central nervous system (CNS) infection. Isolates were classified by PCR detection of *cnf1* or *cnf2*, and only CNF-positive isolates were screened for eight accessory virulence genes. In age-adjusted models restricted to calves aged 2–19 days, overall CNF positivity (aOR 2.83, 95% CI 1.25–6.44; Holm-adjusted *p* = 0.039) and CNF2 positivity (aOR 3.14, 95% CI 1.18–8.31; adjusted *p* = 0.043) were associated with diarrhoea, whereas CNF1 was not. CNF2-positive isolates were enriched for *f17A*, *afa8E*, and *cdtIII*, whereas CNF1-positive isolates more often carried *papC*, *sfa/focDE*, *hlyA*, and *kpsMT* K1. In invasive cases, CNF1 was more frequent in CNS infection than in septicaemia (33.3% vs. 12.7%; adjusted *p* = 0.034). Among CNF1-positive invasive isolates, *kpsMT* K1 occurred in 90.0% of CNS versus 9.1% of septicaemia isolates. These findings support distinct CNF2-associated enteric and CNF1-associated invasive virulence profiles; PCR detection does not establish expression, phenotype, causality, or genomic linkage.

## 1. Introduction

*Escherichia coli* (*E. coli*) is a highly diverse species comprising harmless intestinal commensals as well as strains capable of causing enteric and extraintestinal disease. In calves, *E. coli* can be recovered from clinically healthy animals, diarrhoeic calves, and animals with systemic infection; therefore, isolation alone does not establish a pathogenic role. Interpretation requires consideration of the clinical syndrome, site of isolation, and virulence determinants carried by the isolate. Necrotoxigenic *E. coli* (NTEC) are characterized by cytotoxic necrotizing factors (CNFs), initially recognized through their characteristic effects on cultured cells and subsequently defined at the molecular level [1,2]. CNF1 and CNF2 are the two major *E. coli*-associated variants and form the basis of the conventional distinction between NTEC1 and NTEC2.

CNF1 and CNF2 are closely related single-chain protein exotoxins of approximately 115 kDa. They contain an N-terminal region involved in host-cell binding and membrane translocation and a C-terminal catalytic deamidase domain [3,4], with a Cys-His catalytic centre targeting Rho-family GTPases [5]. Despite their structural similarity, CNF1 and CNF2 generally occur in different genetic backgrounds. *cnf1* is commonly chromosomally encoded in extraintestinal pathogenic *E. coli* (ExPEC), whereas *cnf2* in bovine NTEC2 is typically associated with transmissible Vir or Vir-like plasmids that may also carry genes involved in adhesion, toxin production, and bacterial survival [6,7].

Following cellular uptake [3,4], CNFs modify Rho-family GTPases, which regulate the actin cytoskeleton, cell polarity, migration, and inflammatory signalling. CNF1 deamidates RhoA, Rac1, and Cdc42 [4,8], whereas CNF2 acts particularly strongly on RhoA and Rac1 [9]. Persistent activation of these proteins causes cytoskeletal rearrangement and may alter epithelial barrier function, cell migration, and inflammatory responses [4]. The pathogenic significance of CNFs should therefore be considered within the broader virulence gene profile of the strain, including adhesins, toxins, iron-acquisition systems, and capsule-associated determinants.

In humans, CNF1 is strongly associated with ExPEC, particularly uropathogenic strains, but CNF1-positive isolates have also been described in bacteraemia and neonatal meningitis. These strains frequently carry P fimbriae, S-family adhesins, α-haemolysin, and capsule-associated determinants [4]. Experimental evidence further indicates that CNF1 can facilitate bacterial interaction with the blood–brain barrier [10]. Thus, *cnf1* may occur within a broader ExPEC-like virulence background relevant to invasive infection. In cattle, particularly young calves, CNF2 has been more consistently associated with disease. CNF2-positive NTEC2 have been recovered from calves with diarrhoea and septicaemia [11,12], although they also occur in clinically healthy cattle [13,14]. Experimental infection has shown that bovine NTEC2 can colonize the intestinal tract, induce prolonged diarrhoea, and disseminate beyond the intestine [15]. These strains frequently carry F17 fimbrial genes, Afa-family adhesins, and *cdtIII*, often on Vir/Vir-like plasmids [6,11,16,17]. This combination suggests that the clinical effects of CNF2-positive strains reflect a multicomponent enteric virulence profile rather than CNF2 alone.

Recent studies support interpreting calf-associated *E. coli* according to combinations of virulence determinants rather than individual markers. F17 and other adhesion-associated genes occur in isolates from both diarrhoeic and healthy calves, while broader molecular surveys demonstrate heterogeneous combinations of enteric and extraintestinal virulence genes [18,19,20,21,22,23]. In particular, *iucD*, *f17A*, *afa8E*, and *papC* may coexist within bovine *E. coli* populations [21]. Multi-omics and whole-genome studies further demonstrate substantial diversity in virulence-factor repertoires among calf-associated *E. coli* [24,25], while microbiome studies indicate that diarrhoea also reflects interactions between bacterial traits and the intestinal microbial environment [26]. The molecular characteristics of CNF1-positive *E. coli* in calves remain less well defined, particularly in systemic and central nervous system (CNS) infections. This is relevant because human ExPEC typically possess polygenic virulence repertoires [27]. The accessory markers represent complementary functions: *papC* encodes an usher required for P-fimbrial assembly, *sfa*/*focDE* marks S/F1C fimbrial systems, *f17A* encodes the major F17 fimbrial subunit, and *afa8E* encodes an Afa-VIII afimbrial adhesin [17,27,28,29]. *hlyA* encodes pore-forming α-haemolysin, *cdtIII* marks the type III cytolethal distending toxin locus, *iucD* is involved in biosynthesis of the iron-chelating siderophore aerobactin, and *kpsMT* K1 is a capsule-associated PCR marker [6,27,28,29]. The K1 polysialic acid capsule is particularly associated with invasive disease: group 2 capsules enhance resistance to innate host defences [30], and K1 occurs across several ExPEC phylogroups and promotes survival in human serum by limiting complement-mediated killing [31]. Regulation of K1 capsule synthesis can also influence invasion of human brain microvascular endothelial cells, although K1 alone is not sufficient to explain blood–brain barrier traversal [32]. These observations raise the question of whether CNF1- and CNF2-positive bovine *E. coli* represent distinct molecular virulence backgrounds associated with different clinical manifestations.

Therefore, the aim of this study was to determine the clinical distribution of CNF1- and CNF2-positive *E. coli* in calves and to characterize their associated virulence gene profiles. We evaluated their associations with diarrhoea, septicaemia, and CNS infection and compared accessory virulence determinants between CNF1-positive and CNF2-positive isolates, with particular attention to the K1 capsule marker in invasive infections. We hypothesized that CNF2-positive isolates would be associated predominantly with an *f17A*/*afa8E*/*cdtIII* enteric gene profile, whereas CNF1-positive isolates would more often carry a virulence-marker profile resembling that described for ExPEC, including *papC*, *sfa/focDE*, *hlyA*, and *kpsMT* K1, particularly in CNS infection.

## 2. Results

### 2.1. Study Population and Analytical Framework

The final dataset comprised 544 calves from which *E. coli* was recovered. The enteric group included 131 clinically healthy and 210 diarrhoeic calves sampled on selected farms. Samples originated from different farms or, when a farm contributed more than one calf, from sampling occasions separated by at least one year. Isolates from internal organs and tissues were obtained from 173 calves with septicaemia and 30 with CNS infection submitted for diagnostic necropsy (Table 1). Median age (interquartile range; Table S1) was 18 (13–22) days in healthy calves, 9 (7–12) days in diarrhoeic calves, 5 (3–7) days in septicaemia cases, and 7.5 (5–9) days in CNS cases. Because the enteric and invasive strains were obtained through different sampling routes, they were analysed separately.

### 2.2. Isolates from Healthy and Diarrhoeic Calves

Among calves with *E. coli*-positive faecal samples, CNF-positive isolates were recovered from 14/131 (10.7%) healthy calves and 65/210 (31.0%) diarrhoeic calves. CNF1 positivity was found in 5/131 (3.8%) and 16/210 (7.6%) calves, respectively, and CNF2 positivity in 9/131 (6.9%) and 49/210 (23.3%) calves (Table 1). In the final dataset, CNF1 and CNF2 positivity were mutually exclusive. These crude frequencies are descriptive because healthy calves were older than diarrhoeic calves.

### 2.3. Age-Adjusted Association of CNF Markers with Diarrhoea

The main analysis of enteric isolates was restricted to the age range shared by healthy and diarrhoeic calves (2–19 days), thereby avoiding extrapolation beyond the observed overlap. It included 284 calves (79 healthy and 205 diarrhoeic); 52 healthy calves older than 19 days and five diarrhoeic calves aged 1 day were excluded. After adjustment for linear and quadratic age terms, overall CNF positivity was associated with diarrhoea (aOR 2.83; 95% CI 1.25–6.44; unadjusted *p* = 0.013; Holm-adjusted *p* = 0.039). CNF2 positivity was also associated with diarrhoea (aOR 3.14; 95% CI 1.18–8.31; unadjusted *p* = 0.021; adjusted *p* = 0.043), whereas CNF1 positivity was not (aOR 1.68; 95% CI 0.44–6.40; unadjusted *p* = 0.449; adjusted *p* = 0.449) (Table 2). In the sensitivity analysis including all 341 strains isolated from faecal samples, the results were similar: overall CNF positivity, aOR 2.93 (95% CI 1.29–6.62; adjusted *p* = 0.030); CNF1 positivity, aOR 1.75 (95% CI 0.46–6.60; adjusted *p* = 0.410); and CNF2 positivity, aOR 3.21 (95% CI 1.22–8.48; adjusted *p* = 0.037).

### 2.4. Virulence Gene Profiles of CNF-Positive Escherichia coli

The second-stage molecular analysis included 155 representative CNF-positive isolates (79 from the enteric isolates and 76 from the invasive diagnostic strains), of which 53 were CNF1-positive and 102 were CNF2-positive. None of these representative isolates carried both *cnf1* and *cnf2*. Seven of the eight accessory virulence markers differed significantly between CNF1-positive and CNF2-positive isolates after Holm correction (Table 3). CNF2-positive isolates more frequently carried *f17A*, *afa8E*, and *cdtIII*, whereas CNF1-positive isolates more frequently carried *papC*, *sfa*/*focDE*, *hlyA*, and *kpsMT* K1. *iucD* was the only marker that did not differ between the two groups (Holm-adjusted *p* = 0.613).

**Figure 1:**
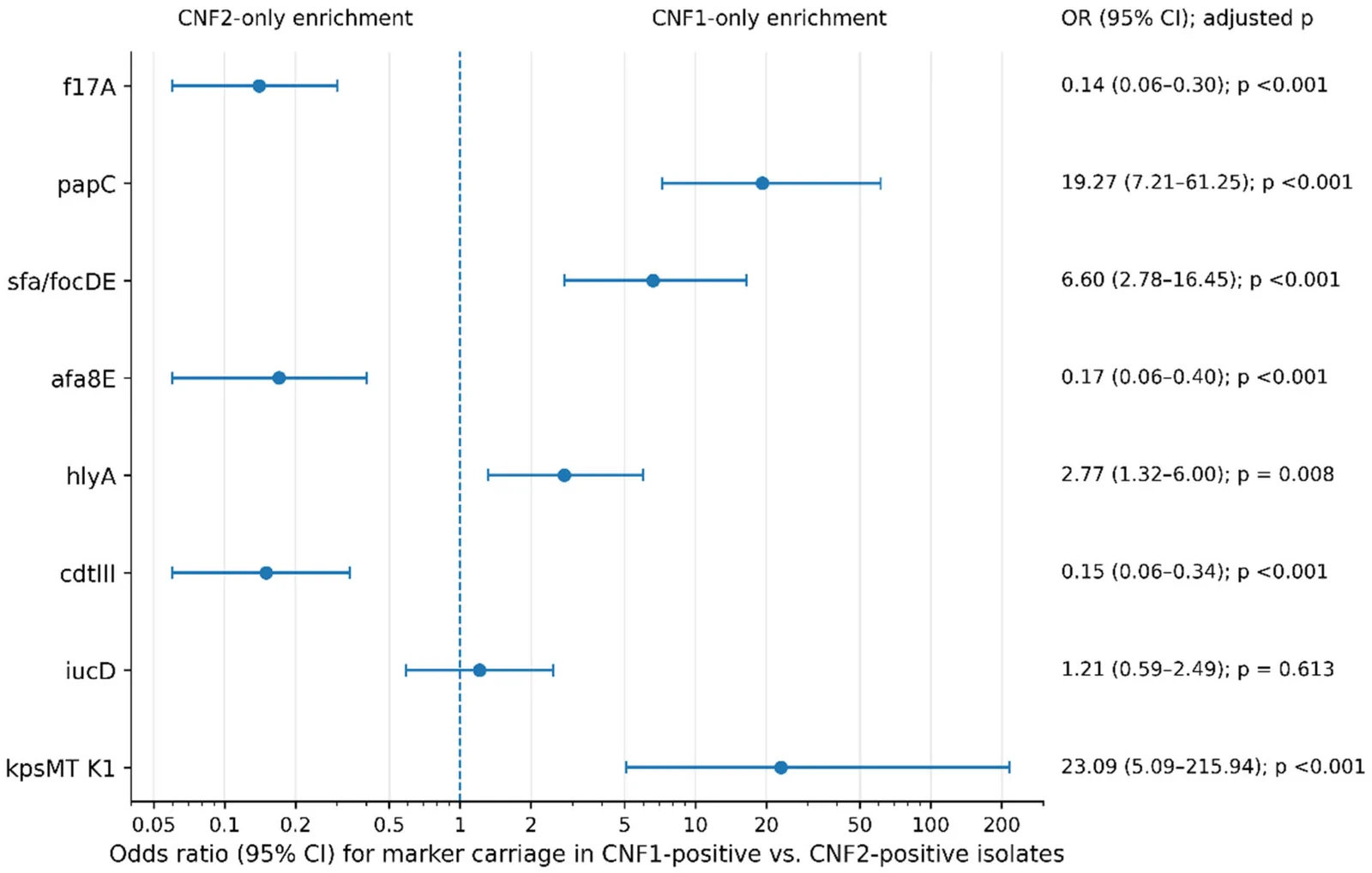
Comparison of accessory virulence genes between CNF1-positive (n = 53) and CNF2-positive (n = 102) *E. coli* isolates. The forest plot is based on PCR-derived gene frequencies presented in Table 3. Points represent conditional maximum-likelihood odds ratios (ORs) with exact 95% confidence intervals (CIs) on a logarithmic scale; *p* values were obtained using two-sided Fisher’s exact tests with Holm correction. OR > 1 indicates enrichment in CNF1-positive isolates; OR < 1 indicates enrichment in CNF2-positive isolates.

The magnitude and direction of these associations are illustrated by the odds ratios and 95% confidence intervals shown in Figure 1. The largest association estimates were observed for *kpsMT* K1 (OR 23.09; 95% CI 5.09–215.94) and *papC* (OR 19.27; 95% CI 7.21–61.25), both enriched in CNF1-positive isolates. *hlyA* showed a smaller but significant association with the CNF1-positive profile (OR 2.77; 95% CI 1.32–6.00; Holm-adjusted *p* = 0.008), whereas *iucD* showed no evidence of differential distribution between CNF1-positive and CNF2-positive isolates.

### 2.5. Isolates from Septicaemia and CNS Infection Cases

The invasive diagnostic series included 173 calves with septicaemia and 30 with CNS infection. CNF positivity was found in 62/173 (35.8%) septicaemia isolates and 14/30 (46.7%) CNS isolates. CNF1 positivity was detected in 22/173 (12.7%) and 10/30 (33.3%) isolates, respectively, whereas CNF2 positivity was detected in 40/173 (23.1%) and 4/30 (13.3%) isolates (Table 4). After Holm correction across the three CNF markers, only CNF1 differed significantly between syndromes (conditional exact OR for CNS infection versus septicaemia 3.40; 95% CI 1.25–8.88; unadjusted *p* = 0.011; adjusted *p* = 0.034). Adjusted *p* values for overall CNF positivity and CNF2 positivity were both 0.616. These frequencies refer to submitted necropsy cases and should not be interpreted as population prevalence estimates.

Among the 76 CNF-positive invasive isolates, *sfa*/*focDE* was detected in 9/14 (64.3%) CNS isolates and 13/62 (21.0%) septicaemia isolates (conditional exact OR 6.57; 95% CI 1.65–29.65; Holm-adjusted *p* = 0.018). *kpsMT* K1 was detected in 10/14 (71.4%) CNS isolates and 2/62 (3.2%) septicaemia isolates (conditional exact OR 65.10; 95% CI 9.86–807.62; adjusted *p* < 0.001). The remaining six accessory markers did not differ significantly after correction. Because the proportions of CNF1- and CNF2-positive isolates differed between syndromes, these comparisons describe the overall CNF-positive invasive subset and do not establish marker-specific effects independent of CNF type (Table 4).

To further evaluate the K1 association within a more homogeneous CNF background, an additional analysis was restricted to CNF1-positive invasive isolates. Within this subset, *kpsMT* K1 was detected in 9/10 (90.0%) CNS isolates and 2/22 (9.1%) septicaemia isolates (conditional exact OR 67.20; 95% CI 5.68–4124.87; two-sided Fisher’s exact *p* < 0.001). The wide confidence interval reflects the small subgroup size. This restricted analysis reduces confounding by CNF type; however, because accessory genes were assessed only among CNF-positive isolates, it does not estimate the independent effect of K1 in the entire invasive *E. coli* population.

## 3. Discussion

Calf health is a major determinant of survival, welfare, and future productivity, with diarrhoea and systemic infections representing important causes of morbidity and mortality during the neonatal period [18,28]. Accurate diagnosis of *E. coli*-associated disease remains challenging because *E. coli* is also a common intestinal commensal, and detection of individual virulence genes does not necessarily establish pathogenicity [20,21]. Consequently, clinical interpretation should integrate disease presentation with the broader virulence profile of the isolate rather than rely on a single molecular marker [27,28]. In the present study, we compared CNF1- and CNF2-positive *E. coli* from calves with enteric and invasive disease and identified distinct virulence-gene patterns associated with diarrhoea, septicaemia, and CNS infection. These findings provide a molecular framework for interpreting the clinical significance of CNF-positive *E. coli* in calves.

### 3.1. CNF2 and Enteric Disease

CNF2 positivity remained associated with diarrhoea after adjustment for age and Holm correction, and the result was similar in the full-range sensitivity analysis. Nevertheless, CNF2-positive isolates were also recovered from healthy calves, as previously reported [13,14]. CNF2 detection should therefore not be interpreted as proof that an individual isolate caused diarrhoea.

This interpretation is consistent with earlier calf studies. Detection of *E. coli*, and even individual diarrhoeagenic virulence genes, does not invariably distinguish diarrhoeic from clinically healthy calves. Ryu et al. identified F17 frequently irrespective of diarrhoeal status, whereas Umpiérrez et al. detected *f17A*, *afa8E*, *papC*, and *iucD* in isolates from both diarrhoeic and asymptomatic calves [20,21]. Other studies have likewise documented heterogeneous combinations of enteric and extraintestinal virulence genes [18,19,22,23]. More broadly, enteric *E. coli* pathogenicity is best interpreted as the result of combinations of adhesins, toxins, and fitness determinants rather than a single marker [28].

The higher frequencies of *f17A*, *afa8E*, and *cdtIII* among CNF2-positive isolates are consistent with classical bovine NTEC2/Vir-like plasmid profiles [6,11,16,17]. In the present study, however, these markers define an associated gene pattern rather than a demonstrated functional module because PCR does not assess gene expression, toxin production, or physical linkage among genes. Clinical interpretation should therefore consider calf age, clinical presentation, and the accompanying virulence gene profile.

### 3.2. Distinct Virulence Backgrounds of CNF1- and CNF2-Positive Isolates

A central finding of this study was the clear separation of accessory virulence gene profiles between CNF1-positive and CNF2-positive isolates. *papC*, *sfa/focDE*, *hlyA*, and *kpsMT* K1 were enriched in CNF1-positive isolates, whereas *f17A*, *afa8E*, and *cdtIII* were enriched in CNF2-positive isolates. This pattern is consistent with CNF1-associated profiles containing selected ExPEC-associated markers and bovine NTEC2-associated enteric backgrounds.

The CNF1-associated profile resembles virulence gene sets described in ExPEC [27]. ExPEC-related determinants have also been detected in *E. coli* from neonatal calves with extraintestinal infections [33], and earlier bovine NTEC studies documented distinct combinations of Pap-, Sfa-, F17-, Afa8-, and CDT-III-associated determinants in isolates from diarrhoeic and septicaemic calves [12]. In the present study, however, the observed similarity to ExPEC is restricted to the selected virulence markers; phylogroup, sequence type, clonality, invasive phenotype, and formal ExPEC classification were not determined. Phylogenetic analysis, MLST, and whole-genome sequencing were not performed.

### 3.3. CNF1, K1 Capsule, and CNS Infection

In the invasive isolates, CNF1 was more frequent in CNS infection than in septicaemia after Holm correction. This comparison concerns recorded diagnostic categories and does not establish distinct routes of infection. Among all CNF-positive invasive isolates, *sfa/focDE* and *kpsMT* K1 were also more frequent in CNS cases. These overall accessory-marker comparisons should be interpreted cautiously because the CNS and septicaemia groups differed in their proportions of CNF1- and CNF2-positive isolates. Importantly, the K1 difference persisted in the targeted analysis restricted to CNF1-positive isolates, in which *kpsMT* K1 was detected in 90.0% of CNS isolates and 9.1% of septicaemia isolates. The very wide confidence interval reflects the small subgroup and warrants caution when interpreting the association estimate. This comparison involved only 10 CNF1-positive CNS isolates and 22 CNF1-positive septicaemia isolates; consequently, the precision of the K1 association estimate is limited, and validation in larger, independent calf populations is warranted.

This finding is biologically plausible because group 2 capsules contribute to resistance against innate host defences, and K1 is widely associated with invasive *E. coli* [30,31,32]. The K1-specific PCR assay used here has been evaluated against serologically determined K antigens [29]. Nevertheless, detection of *kpsMT* K1 does not demonstrate capsule expression, and the K1 marker should not be interpreted as a stand-alone mechanism of CNS invasion. Mechanistic evidence from human or experimental systems cannot be transferred directly to calves.

The data therefore support an association between a CNF1/K1-associated molecular profile and bovine CNS infection, but they do not establish a causal pathway. Functional studies assessing capsule expression, serum resistance, endothelial interaction, and CNF1 activity would be required to test this hypothesis.

The overlap between the CNF1-associated profile identified here and virulence repertoires described in human ExPEC is relevant from a One Health perspective, but shared virulence genes do not demonstrate zoonotic transmission, host sharing, or clonal relatedness. Such conclusions require comparative whole-genome sequencing, phylogenetic analysis, and characterization of mobile genetic elements in appropriate bovine and human reference collections.

Overall, the data support two clinically distinct patterns: CNF2-positive isolates were associated with diarrhoea and an *f17A*/*afa8E*/*cdtIII* gene profile, whereas CNS infection was enriched for CNF1 and, within CNF1-positive invasive isolates, for the K1 capsule marker.

Several limitations should be considered. Healthy and diarrhoeic calves were sampled from farms, whereas invasive cases were diagnostic necropsy submissions; therefore, frequencies between these groups should not be interpreted as population prevalence estimates. Restricting the primary enteric analysis to calves aged 2–19 days improved age comparability but excluded 57 calves, although the full-range sensitivity analysis yielded similar results. Accessory virulence genes were examined only among CNF-positive isolates; consequently, their associations cannot be assessed independently of CNF status in the broader calf *E. coli* population. In the invasive strains, accessory-marker comparisons may also partly reflect the different proportions of CNF1- and CNF2-positive isolates in CNS and septicaemia cases. Multivariable modelling of the invasive strains was limited by the small number of CNS cases and sparse subgroup data, and residual confounding by age and other animal- or farm-level factors cannot be excluded. Farm-level clustering was not incorporated into the models, and some residual within-farm dependence may therefore remain. Other major enteric pathogens and ETEC-associated determinants were not systematically assessed, so co-infections and alternative causes of diarrhoea cannot be excluded. Screening five colonies per faecal sample may have failed to detect low-abundance CNF-positive subpopulations. In addition, PCR detects the presence of selected genes but does not establish their expression, phenotype, genomic linkage, or functional contribution to disease. Representative amplicons were not sequence-confirmed, and phylogroup determination, MLST, and whole-genome sequencing were not performed; thus, some observed differences between CNF1- and CNF2-positive isolates may reflect underlying bacterial lineage structure rather than independent effects of individual virulence determinants.

## 4. Materials and Methods

### 4.1. Study Population and Diagnostic Classification

This observational study included 544 calves from Poland from which *E. coli* was recovered. Healthy and diarrhoeic calves were sampled from the same farms during the 2015–2025 study period. The enteric *E. coli* isolates were obtained from calves with reported diarrhoea on 114 farms, whereas isolates from septicaemia cases were obtained from calves on 130 farms across Poland. The age distribution of calves according to clinical category is presented in Supplementary Table S1. Healthy and diarrhoeic calves were sampled on the same farms. Calves were enrolled on the basis of predefined clinical eligibility criteria, and animals meeting these criteria and available at the time of sampling were included irrespective of subsequent molecular findings. Only calves that had not received prior antimicrobial treatment were included in the study.

Samples originated from different farms or, when one farm contributed more than one calf, from sampling occasions separated by at least one year. Healthy calves were defined by normal faecal consistency and the absence of gastrointestinal signs at sampling, whereas diarrhoeic calves passed loose, semi-liquid, or watery faeces. The invasive group comprised diagnostic necropsy submissions from 173 calves with septicaemia and 30 with CNS infection. Carcasses were refrigerated after death and examined within 24 h. Septicaemia was defined by gross lesions compatible with systemic infection together with recovery of *E. coli* from diagnostically relevant internal sites [34]. CNS infection was defined by macroscopic inflammatory lesions of the central nervous system at necropsy together with recovery of *E. coli* from cerebrospinal fluid, brain, or meningeal samples. A history of neurological signs was recorded when available but was not required for classification. CNS cases could also have concurrent or antecedent septicaemia because CNS infection in calves is commonly septicaemic in origin. This category denotes CNS involvement and does not distinguish primary CNS infection from secondary spread during septicaemia. A subset of the invasive cases overlapped with the previously published necropsy cohort [34]. However, the present study addressed a distinct molecular objective focused on CNF1/CNF2 status and associated virulence profiles, which were not evaluated in the previous study. Eligibility was determined solely from clinical and diagnostic criteria. Clinical classification was completed before molecular results were available, thereby preventing molecular findings from influencing assignment to clinical groups. The calf was the analytical unit and contributed no more than one representative isolate to each analysis. The study was conducted within the framework of a research protocol approved by the Research Ethics Committee of the Wroclaw University of Environmental and Life Sciences (No. NON00000.0020.1.4.16.2026).

### 4.2. Bacteriology and Molecular Characterization

Faecal specimens were streaked onto MacConkey agar and incubated aerobically at 37 °C for 18–24 h. Five spatially separated lactose-positive colonies with morphology consistent with *E. coli* were subcultured and confirmed by routine biochemical identification. Several colonies were examined to account for within-calf *E. coli* heterogeneity, as in previous calf studies [21,35]. Post-mortem blood and samples from the liver, spleen, lungs, brain, cerebrospinal fluid, and meninges were collected aseptically and processed by routine aerobic bacteriology. One representative *E. coli* isolate from a diagnostically relevant site was retained per invasive case. Bacterial DNA was prepared from overnight broth cultures by boiling extraction [36]. Each faecal colony was screened separately for *cnf1* and *cnf2* using published target-specific PCR methods [36,37]. One CNF-positive colony per calf was selected for second-stage characterization. When several colonies had the same CNF genotype, one was selected at random. These isolates were tested for *f17A*, *papC*, *sfa/focDE*, *afa8E*, *hlyA*, *cdtIII*, *iucD*, and *kpsMT* K1 using published PCR assays [29,36,38]. The K1 assay used the type-specific *kpsMT* K1 primer paired with the *kpsII* reverse primer described by Johnson and Stell, with an expected amplicon of 153 bp [29]. Positive controls appropriate for each target and no-template negative controls were included. Amplicons were resolved by agarose-gel electrophoresis. Primer sequences and expected amplicon sizes are provided in Table S2, and PCR reaction and electrophoresis conditions are provided in Table S3. Throughout the manuscript, “CNF1-positive” and “CNF2-positive” denote PCR detection of *cnf1* and *cnf2*, respectively; toxin expression was not assessed.

### 4.3. Statistical Analysis

Categorical data were summarized as counts and percentages, whereas age was summarized as median and interquartile range. The enteric and invasive strains were analysed separately. Analyses treated calves as independent observations and did not account for clustering within farms. The primary enteric analysis included healthy and diarrhoeic calves aged 2–19 days (n = 284), corresponding to the shared observed age range of the two groups. An analysis including all 341 enteric calves was performed as a sensitivity analysis. Separate logistic regression models used diarrhoea as the binary outcome and overall CNF, CNF1, or CNF2 positivity as the exposure of interest; each model included linear and quadratic age terms. Wald adjusted odds ratios (aORs) with 95% confidence intervals (CIs) were reported, and *p* values were adjusted across the three models using the Holm method. The same multiplicity adjustment was applied in the sensitivity analysis. Multivariable modelling of the invasive strains was considered but was not performed because the limited number of CNS cases and sparse data after stratification would have produced unstable estimates. In the invasive strains, CNF-marker frequencies in CNS infection and septicaemia were compared using two-sided Fisher’s exact tests with Holm adjustment across the three markers. Conditional maximum-likelihood odds ratios (ORs) with exact 95% CIs were used for sparse 2 × 2 tables. Accessory markers were analysed only among CNF-positive isolates. CNF1-positive and CNF2-positive isolates were compared using two-sided Fisher’s exact tests; conditional maximum-likelihood ORs with exact 95% CIs were reported, and *p* values were adjusted across eight markers using the Holm method. Accessory-marker comparisons between CNS infection and septicaemia among CNF-positive invasive isolates used the same procedure with Holm adjustment across eight markers. The targeted *kpsMT* K1 comparison within CNF1-positive invasive isolates used a two-sided Fisher’s exact test and a conditional exact OR with exact 95% CI. For families of multiple comparisons, statistical significance was defined as Holm-adjusted *p* < 0.05; for the targeted within-CNF1 K1 comparison, a two-sided *p* < 0.05 was used. Statistical analyses were performed in R version 4.5.1 (R Foundation for Statistical Computing, Vienna, Austria).

## 5. Conclusions

CNF1- and CNF2-positive *Escherichia coli* differed in their associations with clinical presentation and in their accessory virulence-gene profiles in this calf collection. After adjustment for age and Holm correction, CNF2 positivity was associated with diarrhoea, and CNF2-positive isolates were characterized mainly by the *f17A/afa8E/cdtIII* profile. In contrast, CNF1 was more frequent in CNS involvement than in septicaemia, and CNF1-positive isolates were enriched in *papC*, *sfa/focDE*, *hlyA*, and *kpsMT* K1. Within CNF1-positive invasive isolates, *kpsMT* K1 was markedly more frequent in CNS infection, supporting an imprecisely estimated molecular association that warrants functional and genomic validation. Collectively, these findings indicate that CNF1 and CNF2 should be interpreted within broader virulence-gene backgrounds rather than as isolated markers. Because the study was observational and PCR-based, the results do not establish gene expression, phenotype, independent pathogenic effects, causality, or genomic linkage.

## Figures and Tables

**Table 1:** Distribution of CNF markers among *E. coli*-positive healthy and diarrhoeic calves.

Clinical Group	n	CNF+ n (%)	CNF1+ n (%)	CNF2+ n (%)
Healthy	131	14 (10.7)	5 (3.8)	9 (6.9)
Diarrhoea	210	65 (31.0)	16 (7.6)	49 (23.3)

**Table 2:** Age-adjusted logistic regression for diarrhoea among *E. coli*-positive calves in the overlapping age range (2–19 days).

Bacterial Marker	aOR	95% CI	Raw *p*	Holm-Adjusted *p*
CNF+	2.83	1.25–6.44	0.013	0.039
CNF1+	1.68	0.44–6.40	0.449	0.449
CNF2+	3.14	1.18–8.31	0.021	0.043

Outcome: diarrhoea = 1 and healthy = 0. Separate logistic regression models used diarrhoea as the outcome and one of three related CNF-marker exposures (overall CNF, CNF1, or CNF2 positivity) as the predictor of interest; all models included linear and quadratic age terms; *p* values were adjusted across the three models using the Holm procedure. aOR, adjusted odds ratio; CI, confidence interval.

**Table 3:** Accessory virulence markers among CNF1-positive and CNF2-positive *Escherichia coli* isolates.

Marker	CNF1+ (n = 53), n (%)	CNF2+ (n = 102), n (%)	OR (95% CI)	Holm-Adjusted *p*
*f17A*	14 (26.4)	74 (72.5)	0.14 (0.06–0.30)	<0.001
*papC*	47 (88.7)	29 (28.4)	19.27 (7.21–61.25)	<0.001
*sfa/focDE*	25 (47.2)	12 (11.8)	6.60 (2.78–16.45)	<0.001
*afa8E*	8 (15.1)	53 (52.0)	0.17 (0.06–0.40)	<0.001
*hlyA*	36 (67.9)	44 (43.1)	2.77 (1.32–6.00)	0.008
*cdtIII*	11 (20.8)	65 (63.7)	0.15 (0.06–0.34)	<0.001
*iucD*	27 (50.9)	47 (46.1)	1.21 (0.59–2.49)	0.613
*kpsMT* K1	17 (32.1)	2 (2.0)	23.09 (5.09–215.94)	<0.001

ORs are conditional maximum-likelihood estimates with exact 95% CIs from two-sided Fisher’s exact tests; *p* values were adjusted across eight markers using the Holm procedure. OR > 1 indicates a higher marker frequency in CNF1-positive isolates; OR < 1 indicates a higher frequency in CNF2-positive isolates. CI, confidence interval; OR, odds ratio.

**Table 4:** Distribution of CNF markers among *E. coli*-positive invasive cases.

Syndrome	n	CNF+ n (%)	CNF1+ n (%)	CNF2+, n (%)
Septicaemia	173	62 (35.8)	22 (12.7)	40 (23.1)
CNS infection	30	14 (46.7)	10 (33.3)	4 (13.3)

Percentages use *E. coli*-positive invasive cases as the denominator. Comparing CNS infection with septicaemia, conditional exact ORs (95% CIs), unadjusted *p* values, and Holm-adjusted *p* values were: overall CNF positivity, 1.56 (0.66–3.68), 0.308, 0.616; CNF1 positivity, 3.40 (1.25–8.88), 0.011, 0.034; and CNF2 positivity, 0.51 (0.12–1.61), 0.337, 0.616. The *p* values were obtained using two-sided Fisher’s exact tests and adjusted across the three markers. CNF1+ and CNF2+ were mutually exclusive in the final dataset.

## Data Availability

The raw data supporting the conclusions of this article will be made available by the corresponding author on request.

## Supplementary Materials

The following supporting information can be downloaded at: https://www.mdpi.com/article/10.3390/ijms27219408/s1.

## Abbreviations

The following abbreviations are used in this manuscript:

aOR	adjusted odds ratio
CDT-III	cytolethal distending toxin type III
CI	confidence interval
CNF	cytotoxic necrotizing factor
CNF1	cytotoxic necrotizing factor type 1
CNF2	cytotoxic necrotizing factor type 2
CNS	central nervous system
ExPEC	extraintestinal pathogenic *Escherichia coli*
NTEC	necrotoxigenic *Escherichia coli*
NTEC1	type 1 necrotoxigenic *Escherichia coli*
NTEC2	type 2 necrotoxigenic *Escherichia coli*
OR	odds ratio
PCR	polymerase chain reaction
