# Supplementary material for: Clinical Distribution and Virulence Gene Profiles of CNF1- and CNF2-Positive Escherichia coli in Calves
Source: Int J Mol Sci. 2026 Oct 22;27(21):9408. doi: 10.3390/ijms27219408 (PMC13763134; doi:10.3390/ijms27219408)
Supplement: Supplementary file 1 [file ijms-27-09408-s001.zip › ijms-4593269-supplementary.pdf]

Supplementary Material

Supplementary Table S1. Age distribution of calves according to clinical category

Clinical category	n	Age, median (IQR), days
Healthy	131	18 (13–22)
Diarrhoea	210	9 (7–12)
Septicaemia	173	5 (3–7)
CNS infection	30	7.5 (5–9)

IQR, interquartile range; CNS, central nervous system.

Supplementary Table S2. Primer sequences, expected amplicon sizes, and source PCR assays

Target gene / marker	Primer	Primer sequence (5′→3′)	Amplicon (bp)	Source
cnf1	CNF1-A	GAACTTATTAAGGATAGT	543	Blanco et al. [37]
	CNF1-B	CATTATTTATAACGCTG		
cnf2	CNF2-A	AATCTAATTAAAGAGAAC	543	Blanco et al. [37]
	CNF2-B	CATGCTTTGTATATCTA		
f17A	F	GCAGAAAATTCAATTTATCCTTGG	537	Van Bost et al. [36]
	R	CTGATAAGCGATGGTGTAATTAAC		
papC	F	GACGGCTGTACTGCAGGGTGTGGCG	328	Van Bost et al. [36]
	R	ATATCCTTTCTGCAGGGATGCAATA		
sfa/focDE (sfaD-E region)	F	CTCCGGAGAACTGGGTGCATCTTAC	410	Van Bost et al. [36]
	R	CGGAGGAGTAATTACAAACCTGGCA		
afa8E	F	CTAACTTGCCATGCTGTGACAGTA	302	Van Bost et al. [36]
	R	TTATCCCCTGCTTAGTTGTGAATC		
hlyA	F	AACAAGGATAAGCACTGTTCTGGCT	1177	Johnson and Stell [29]
	R	ACCATATAAGCGGTCATTCCCGTCA		
cdtIII	F	GAAAATAAATGGAATATAAATGTCCG	555	Van Bost et al. [36]
	R	TTTGTGTCGGTGCAGCAGGGAAAA		Bihannic et al. [38]
iucD	F	AAAACTGACATCGGATGGC	253	Van Bost et al. [36]
	R	GTATTTGTGGCAACGCAGAA		
kpsMT K1	K1-F	TAGCAAACGTTCTATTGGTGC	153	Johnson & Stell [29]
	kpsII-R	CATCCAGACGATAAGCATGAGCA		

Notes. Positive-control strains used in the study had previously been confirmed as positive for the corresponding target genes by sequencing.

Supplementary Table S3. PCR reaction conditions and electrophoresis conditions used for the published assay systems

Assay / target genes	Reaction mixture	Amplification program	Source
CNF1/CNF2 type-specific PCR (cnf1, cnf2)	25 μL per singleplex reaction: 2.5 μL 10× PCR buffer, 2.5 mM $MgCl_2$, 1.0 μL dNTP Mix (10 mM each dNTP; final concentration 400 μM each), 1.25 U Taq DNA polymerase, 5.8 μL template DNA, 0.8 μL forward primer + 0.8 μL reverse primer (90 ng/μL stock; either the cnf1 pair or the cnf2 pair), and nuclease-free water to 25.0 μL.	Initial denaturation 94 °C, 5 min; 30 cycles of 94 °C, 1 min; 43 °C, 1 min; 72 °C, 2 min; final extension 72 °C, 7 min.	Blanąco et al. [37]
Van Bost M1 (cdtIII, iucD)	25 μL total volume containing 1.0 μL boiled-lysate supernatant, 0.25 μL of each primer, 2.5 μL dNTP Mix (10 mM each dNTP), 2.5 μL PCR buffer, 1.25 U Taq DNA polymerase, and nuclease-free water to 25 μL.	Initial denaturation 94 °C, 3 min; 30 cycles of 94 °C, 1 min; 60 °C, 1 min; 72 °C, 1 min; final extension 72 °C, 7 min.	Van Bost et al. [36]
Van Bost M2 (f17A, papC, sfa/focDE, afa8E)	25 μL total volume containing 1.0 μL boiled-lysate supernatant, 0.25 μL of each primer, 2.5 μL dNTP Mix (10 mM each dNTP), 2.5 μL PCR buffer, 1.25 U Taq DNA polymerase, and nuclease-free water to 25 μL.	Initial denaturation 95 °C, 5 min; 30 cycles of 95 °C, 1 min; 57 °C, 1 min; 72 °C, 1 min; final extension 72 °C, 7 min.	Van Bost et al. [36]
Johnson–Stell multiplex PCR system (hlyA; kpsMT K1)	25 μL total volume containing 2 μL boiled lysate, 4 mM $MgCl_2$, 2.0 μL dNTP Mix (10 mM each dNTP; final concentration 0.8 mM each), 0.6 μM of each primer, 2.5 U Taq DNA polymerase, and 1× PCR buffer. (The 0.3 μM exception in the source applies only to primer pairs marked with an asterisk; hlyA and K1 are not in that category.)	AmpliTaq Gold activation 95 °C, 12 min; 30 cycles of 94 °C, 30 s; 63 °C, 30 s; 68 °C, 3 min; final extension 72 °C, 10 min.	Johnson and Stell [29]
